# Weight reduction does not induce an undesirable decrease in muscle mass, muscle strength, or physical performance in men with obesity: a pilot study

**DOI:** 10.20463/jenb.2017.0029

**Published:** 2017-12-31

**Authors:** Bokun Kim, Takehiko Tsujimoto, Rina So, Xiaoguang Zhao, Sechang Oh, Kiyoji Tanaka

**Affiliations:** 1. Faculty of Sports Health Care, Inje University, Gimhae, 50834 Republic of Korea; 2. Foundation for Industry-Academy Cooperation, Dong-A University, Pusan Republic of Korea; 3. Faculty of Human Sciences, Shimane University, Shimane Japan; 4. Faculty of Health and Sport Sciences, University of Tsukuba, Ibaraki Japan; 5. Research Center for Overwork-Related Disorders, National Institute of Occupational Safety and Health, Kanagawa Japan; 6. Faculty of Sports Science, Ningbo University, Zhejiang China; 7. Faculty of Medicine, University of Tsukuba, Ibaraki Japan; 8. The Center of Sports Medicine and Health Sciences, University of Tsukuba Hospital, Ibaraki Japan

**Keywords:** Muscle mass, muscle strength, musculoskeletal condition, obesity, physical performance, weight reduction

## Abstract

**[Purpose]:**

To date, there have been no reports on whether weight reduction causes decreases in muscle mass, muscle strength, or physical performance that could lead to health problems. Thus, in this pilot study, we investigated the appropriateness of the changes in muscle mass, muscle strength and physical performance after weight reduction.

**[Methods]:**

Obese men who completed a weight reduction program to decrease and maintain a body mass index (BMI) of less than 25 kg/m2 for one year were recruited for the study. One year after the completion of a weight reduction program, the participants’ muscle mass, muscle strength, and physical performance were compared with those in a reference group composed of individuals whose BMI was less than 25 kg/m2. Whole-body scanning was performed using dual-energy X-ray absorptiometry to analyze muscle mass. Handgrip strength and knee extensor strength were measured to evaluate arm and leg muscle strength, respectively. For physical performance, a jump test was employed.

**[Results]:**

The results showed that the biceps, triceps, subscapular, and suprailiac areas of professional fashion models were significantly thinner than those of women in general (p<.001), and that their waist size was also significantly smaller (p<.001). However, hip circumference showed no significant difference. Body mass index, waist-to-hip ratio, and body fat (%) in professional fashion models were significantly lower than those in women in general (p<.001), while the body density in professional fashion models was significantly greater (p<0.001).

**[Conclusion]:**

Weight reduction participants showed an average reduction in body weight of -16.47%. Normalized arm muscle mass and handgrip strength were significantly greater in the weight reduction group than in the reference group; however, no significant differences were detected between the two groups with respect to the other variables. After one year, there were no significant differences between the two groups.

## INTRODUCTION

Obesity causes a broad range of chronic conditions, including heart disease, hypertension, and diabetes ^1^, and contributes to musculoskeletal conditions, such as osteoarthritis, epicondylitis, tendinitis, and back pain ^2^^-^^4^. The incidence of osteoarthritis increases by 36% for every 5-kg increase in body weight ^5^. A 5% reduction in body weight decreases joint pain, and a 10% reduction in body weight is associated with moderate-to-considerable clinical improvement in joint pain ^6^. Reducing the body mass index (BMI) to 20–24.9 kg/m2 can decrease osteoarthritis in more than 50% of cases ^7^. Thus, weight reduction is beneficial in preventing or ameliorating musculoskeletal conditions. 

Although the effect of weight reduction on musculoskeletal conditions is clear, weight reduction decreases both fat mass and muscle mass ^8^. Muscle strength is positively associated with muscle mass. A rapid decrease in muscle mass caused by substantial weight reduction might decrease muscle strength, which is likely to decrease physical performance ^8^^-^^11^. Low muscle mass and strength are linked to the incidence of musculoskeletal conditions ^10^^,^ ^12^^-^^14^. The concomitant loss of muscle mass and strength induced by weight reduction is, therefore, an important concern. 

In our previous study, significant decreases in both muscle mass and muscle strength after weight reduction were observed ^8^^,^ ^9^. However, we could not determine whether these changes were desirable without further investigation. To the best of our knowledge, there have been no reports on whether weight reduction leads to detrimental decreases in muscle mass, muscle strength, or physical performance that could lead to health problems. The lack of research on this issue reflects a failure to appreciate the importance of the effects of weight reduction on muscle mass and strength. Thus, in this pilot study, we investigated the appropriateness of the changes in muscle mass, muscle strength, and physical performance after weight reduction. 

## METHODS

### Study design and analysis subjects

In this pilot study, a one-year follow-up assessment of a prospective study was performed to investigate the appropriateness of the changes in muscle mass and strength induced by a weight reduction program comprising caloric restriction and exercise. The details of our previous study have been reported ^8^^,^ ^9^. Briefly, 97 men with obesity were involved in a 12-week weight reduction program from May to July 2012, and 2013 at the University of Tsukuba, Japan. In the caloric restriction class, the subjects were instructed to consume approximately 1680 kcal/ day. This program was based on the Four-Food-Group Point Method, which divides the diet into the following four food groups based on nutritional content: Group 1 (dairy products and eggs), Group 2 (beans, fish, and meat), Group 3 (fruits and vegetables), and Group 4 (sugar and grains). For nutrient balance calculations and assessments of energy intake, all foods were portioned into 80-kcal servings, and each portion was regarded as 1 point. For each meal, the subjects were instructed to select 1, 2, 1, and 3 points of diverse foods from food groups 1, 2, 3, and 4, respectively, to consume a well-balanced daily diet. The subjects participated in a 90-minute combined aerobic exercise program 3 days/week for 12 weeks. Each class began with 10–20 minutes of warm-up activities such as stretching. These activities were followed by the main exercise: 40–60 minutes of brisk walking and jogging outdoors. On rainy days, indoor exercise using stationary cycling, and ladder climbing were the main exercises. Each class concluded with 10–20 minutes of resistance exercise using the body weights of the subjects, and cool-down exercises. All assessments were conducted before the start of the weight reduction program and were repeated within 2 weeks after the last session of the weight reduction program. For the final analysis, 37 participants were excluded owing to dropout, data deficits, or lack of participation in the assessment. The remaining 60 participants were analyzed and described in our previous study, which reported that the weight reduction program caused a 14.1% weight reduction accompanied by an independent decrease in muscle mass and strength. 

**Figure 1.**
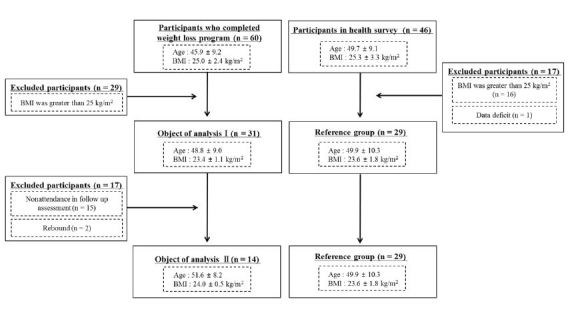
Flow chart of the study subjects.

To investigate the appropriateness of the changes in muscle mass, muscle strength, and physical performance after weight reduction, we compared the physical parameters of the participants of the weight reduction program with those of a reference group at the completion of the weight reduction program and at one year after completion of the weight reduction program. The Japanese obesity guidelines define obesity as a BMI ≥25 kg/m2 ^15^. Based on this guideline, participants whose BMI decreased to less than 25 kg/m2 during the weight reduction program and who maintained their BMI at less than 25 kg/m2 one year after the weight reduction program were defined as the analysis subjects. As shown in Figure 1, 31 subjects were included as analysis subjects at the completion of the weight reduction program. For data collection at the one-year follow-up, we sent study flyers to the 31 subjects to notify them of the follow-up assessment and to survey their participation in the follow-up assessment 11 months after completing the weight reduction program. We performed the follow-up assessment at the beginning of August in 2013 and 2014. Of the 31 subjects, 17 were excluded from the final analysis. As one of the purposes of the study was health support in the region, we could not force the participants to attend the follow-up assessment. In total, 14 subjects were included as analysis subjects for the one-year follow-up. 

We conducted a health survey to collect data for the reference group in April 2014 at the University of Tsukuba, Japan. The subjects for the survey were recruited from the community through an advertisement in a local information magazine. We adopted the following eligibility criteria for participation in the survey: men aged 30–64 years without terminal disease, recent muscle injury, or surgery. As shown in Figure 1, 46 men participated in the survey, and 29 subjects with a BMI <25 kg/m2 were included as the reference group. All the subjects agreed to participate in the study and provided written informed consent. The study protocol was approved by the Institutional Review Board of the University of Tsukuba, and it met the standards of the Declaration of Helsinki guidelines. 

### Anthropometric and body composition

Height and body weight of the subjects were measured to the nearest 0.1 cm or 0.1 kg, respectively, with the subjects wearing light garments. BMI was computed as the weight divided by the height squared (kg/m2). Wholebody dual-energy X-ray absorptiometry (DXA; QDR 4500; Hologic, Inc., Bedford, MA, USA) was used to measure body composition, as described previously ^16^. We computed the appendicular skeletal muscle mass of each subject as the sum of the lean mass, excluding the bone mineral content, of the upper and lower extremities. A height-adjusted index was then computed by dividing the appendicular skeletal muscle mass in kg of each subject by the square of his height in meters squared (m2) ^17^^-^^20^. We defined the height-adjusted appendicular skeletal muscle index as the skeletal muscle mass index (SMI). The percentage of muscle mass index (%MMI) was computed by dividing the appendicular skeletal muscle mass in kg of each subject by his body weight and multiplying the result by 100 ^21^. 

### Muscle strength

For the upper and lower extremities, we employed handgrip strength and knee extensor strength, respectively, to assess muscle strength. Handgrip strength has been broadly employed to evaluate muscle strength due to its ease of assessment. Measuring knee extensor strength is especially important because it evaluates the quadriceps muscle at the most frequent region of musculoskeletal conditions ^5^^,^ ^22^^,^ ^23^. These types of muscle strength were assessed as follows. 

### Handgrip strength

The subjects were asked to stand straight with their head up, and hold a dynamometer (Grip-D, T.K.K. 5401; TAKEI, Tokyo, Japan) in each hand without putting any pressure on the dynamometer. The subjects were told to fully extend their elbows and simultaneously exert maximum force on the dynamometer ^24^. The assessment was performed twice in each hand, and the highest score result was adopted. This score was represented as an absolute, body weight-normalized, and arm muscle mass-normalized value. 

### Knee extensor strength

 Isometric and isokinetic knee extensor strength were assessed using a Biodex System 3 dynamometer (Biodex Medical Systems, Shirley, NY, USA). Isometric knee extensor strength was mea-sured with the knee at 60°, because this angle provides close-to-optimum muscle lengths for the quadriceps to produce maximal force ^25^. The protocol for the isometric assessment consisted of three maximal extension efforts, each lasting 3 seconds, with 15-second intervening pauses. The isokinetic assessment comprised three maximal extensions at an angular velocity of 60°/s, as is broadly employed for isokinetic muscle strength evaluations ^8^^,^ ^9^^,^ ^11^^,^ ^16^. The highest muscular force output at any moment during the assessment was defined as the peak torque, and was reported in absolute terms (Nm) and normalized to the body weight, represented as the body weight-normalized (Nm/kg) peak torque. We employed the peak torque in the isometric assessment to evaluate static maximal muscle strength, peak torque in the isokinetic assessment to evaluate dynamic maximal muscle strength, amount of work in the isokinetic assessment to evaluate dynamic muscle endurance, and average power in the isokinetic assessment to evaluate dynamic muscle power. The amount of work accomplished in an entire assessment was defined as the total work and was represented as an absolute value (J), whereas the average power was defined as the average of the total work divided by time and was represented as an absolute value (W). All assessments were performed on each leg, and the lower-extremity muscle strength was computed as the average strength in both legs ^8^^,^ ^9^. 

### Physical performance

We selected the jump test, as it directly demonstrates the body weight and lower-extremity muscle strength of a subject. The subjects were asked to stand on a circular board with a dynamometer (Jump-MD; T.K.K. 5106; TAKEI, Tokyo, Japan) wound around the waist. The subjects leapt vertically as high as possible, using a knee countermovement and landing on the circular board of the dynamometer. The assessment was conducted twice, and the highest score was considered. 

### Statistical analysis

Data were analyzed using SPSS software (version 20.0; IBM, Inc., Armonk, USA). The paired t-test was employed to assess differences between the variables before and after the program. The independent-sample t-test was employed for normally distributed data. Otherwise, the Mann–Whitney U test was employed. Data are expressed as the means ± standard deviation or as the means ± standard error. P < 0.05 was considered statistically significant. Cohen’s d was adopted to calculate the effect size. 

## RESULTS

Table 1 shows the characteristics of the subjects in the anthropometric, body composition, muscle strength, and physical performance analyses (Analysis I) before and after participating in a 12-week weight reduction program. During the program, the subjects showed an average reduction in body weight of −13.74 ± 3.75 kg (−16.47 ± 3.79%, P < 0.01). After weight reduction, significant decreases in body composition were detected. The percentages of whole body fat, arm muscle mass (AMM), leg muscle mass (LMM), SMI, and %MMI were significantly reduced (P < 0.01 for all). 

**Table 1:** Characteristics of the subjects in the anthropometric and body composition analyses (Analysis I), and the differences between the two groups before and after a 12-week weight reduction program.

	Pre (range)	Post (range)	Change (95% CI)	P
Age, year	48.81 ± 8.95 (31.00, 63.00)			
Height, cm	171.83 ± 5.15 (161.20, 180.00)			
Weight, kg	82.76 ± 6.53 (70.30, 96.10)	69.03 ± 5.01 (60.40, 79.00)	-13.74 ± 3.75 (-15.11, -12.36)	< 0.01
BMI, kg/m^2^	28.01 ± 1.44 (26.20, 31.34)	23.36 ± 1.06 (21.42, 24.89)	-4.64 ± 1.21 (-5.09, -4.20)	< 0.01
% whole body fat, kg	23.98 ± 4.00 (19.61, 33.26)	18.27 ± 4.74 (11.85, 29.03)	-5.71 ± 2.08 (-6.45, -4.97)	< 0.01
Arm muscle mass, kg	6.46 ± 0.70 (5.11, 7.97)	5.00 ± 4.74 (2.63, 6.61)	-1.48 ± 0.78 (-1.76, -1.21)	< 0.01
Leg muscle mass, kg	19.44 ± 1.77 (16.81, 23.15)	18.12 ± 1.56 (15.79, 22.31)	-1.32 ± 0.73 (-1.58, -1.05)	< 0.01
SMI, kg/m^2^	8.77 ± 0.58 (7.50, 10.06)	7.83 ± 0.56 (7.01, 9.16)	-0.94 ± 0.34 (-1.06, -0.81)	< 0.01
% MMI, %	31.31 ± 1.65 (27.82, 34.90)	33.55 ± 2.25 (29.83, 38.17)	2.24 ± 1.74 (1.60, 2.87)	< 0.01

NOTES: Mean ± standard deviation (range). Abbreviations: CI = confidence interval; BMI = body mass index; % whole body fat = percentage of whole body fat; SMI = skeletal muscle mass index; % MMI = percentage of muscle mass index.

Table 2 shows the characteristics of the subjects in the muscle strength and physical performance analyses (Analysis I) before and after the 12-week weight reduction program. For muscle strength, the absolute values of handgrip strength did not significantly change, whereas handgrip strength per body weight and AMM (P < 0.01 for both) significantly increased. Static and dynamic maximal leg muscle strength decreased significantly (P < 0.05 for both), whereas static and dynamic maximal leg muscle strength per body weight, and dynamic leg muscle power and endurance per body weight significantly increased (P < 0.01 for all). The following variables did not significantly change: dynamic leg muscle endurance and power, static and dynamic maximal leg muscle strength per LMM, and dynamic leg muscle power and endurance per LMM. With respect to physical performance, no significant changes were detected. 

**Table 2:** Characteristics of the muscle strength and physical performance parameters (Analysis I), and the differences between the two groups before and after a 12-week weight reduction program.

	Pre (range)	Post (range)	Change (95% CI)	P
HGS, kg	42.09 ± 5.65 (31.70, 52.70)	41.68 ± 5.56 (30.45, 50.50)	-0.40 ± 2.50 (-1.32, 0.51)	0.37
HGS/BW, kg	0.51 ± 0.08 (0.38, 0.66)	0.61 ± 0.10 (0.41, 0.77)	0.10 ± 0.05 (0.08, 0.11)	< 0.01
HGS/AMM, kg	6.56 ± 0.89 (5.10, 8.37)	8.46 ± 1.27 (5.84, 11.57)	1.90 ± 1.29 (1.43, 2.37)	< 0.01
IMT60 PTQ, Nm	199.65 ± 40.57 (106.15, 300.00)	185.24 ± 33.27 (108.65, 257.15)	-14.40 ± 29.14 (-25.09, -3.72)	< 0.05
IMT60 PTQ/BW, Nm/kg	2.41 ± 0.43 (1.25, 3.12)	2.69 ± 0.46 (1.53, 3.72)	0.28 ± 0.37 (0.14, 0.42)	< 0.01
IMT60 PTQ/LMM, Nm/kg	10.27 ± 1.77 (5.44, 13.54)	10.24 ± 1.68 (5.83, 13.47)	-0.03 ± 1.59 (-0.61, 0.56)	0.93
IKT60 PTQ, Nm	172.64 ± 37.26 (99.30, 263.75)	160.51 ± 23.23 (128.00, 224.65)	-12.14 ± 25.35 (-21.44, -2.84)	< 0.05
IKT 60 PTQ/BW, Nm/kg	2.08 ± 0.38 (1.21, 2.74)	2.33 ± 0.30 (1.82, 3.17)	0.24 ± 0.29 (0.14, 0.35)	< 0.01
IKT 60 PTQ/LMM, Nm/kg	8.85 ± 1.52 (5.53, 11.91)	8.86 ± 1.00 (6.92, 10.86)	0.01 ± 1.33 (-0.48, 0.50)	0.98
IKT60 TW, J	485.67 ± 103.48 (221.35, 719.50)	460.09 ± 65.79 (351.50, 630.60)	-25.59 ± 79.77 (-54.85, 3.68)	0.08
IKT60 TW/BW, J/kg	5.86 ± 1.10 (2.77, 7.49)	6.67 ± 0.84 (4.97, 8.62)	0.81 ± 0.95 (0.46, 1.16)	< 0.01
IKT60 TW/LMM, J/kg	24.91 ± 4.34 (12.32, 34.44)	25.41 ± 2.98 (18.86, 32.18)	0.49 ± 4.26 (-1.07, 2.06)	0.52
IKT60 AP, W	102.36 ± 25.14 (43.95, 156.50)	100.63 ± 17.03 (74.10, 147.95)	-1.72 ± 15.64 (-7.46, 4.01)	0.54
IKT60 AP/BW, W/kg	1.24 ± 0.28 (0.55, 1.69)	1.46 ± 0.24 (1.11, 2.08)	0.22 ± 0.19 (0.16, 0.29)	< 0.01
IKT60 AP/LMM, W/kg	5.25 ± 1.13 (2.45, 7.56)	5.56 ± 0.80 (4.20, 7.15)	0.30 ± 0.86 (-0.01, 0.29)	0.06
Jump test, cm	43.32 ± 5.99 (32.00, 56.00)	44.16 ± 6.45 (30.00, 56.00)	0.84 ± 5.57 (-2.88, 1.20)	0.41

NOTES: Mean ± standard deviation (range). Abbreviations: CI = confidence interval; HGS = handgrip strength; BW = body weight; AMM = arm muscle mass; IMT60 PTQ = isometric60 peak torque; IKT60 PTQ = isokinetic60 peak torque; Isokinetic60 TW = isokinetic60 total work; IKT60 AP = isokinetic60 average power.

The anthropometric and body composition characteristics as well as the differences between the two groups after completing the weight reduction program are presented in Table 3. Age, height, body weight, and BMI did not significantly differ between the groups. There were no significant differences in the total body fat percentage or leg muscle mass between the groups. With respect to the arm muscle mass, SMI and %MMI, the values in the weight reduction group were significantly lower than those in the reference group (P < 0.05). The arm muscle mass in the weight reduction group contributed to the difference between the groups with regard to SMI and %MMI. 

**Table 3:** Characteristics of subjects in the anthropometric and body composition analyses, and the differences between the two groups after a 12-week weight reduction program.

	Reference group^a^	Weight reduction group^a^	Mean difference^b^	P	Effect size
	(n = 29)	(n = 31)	(95% CI)		
Age, year	49.96 ± 10.45	48.81 ± 8.95	1.16 ± 2.53 (-3.90, 6.22)	0.65	0.118
Height, cm	172.23 ± 5.56	171.83 ± 5.15	0.69 ± 1.40 (-2.12, 3.50)	0.63	0.075
Weight, kg	69.21 ± 6.11	69.03 ± 5.01	0.19 ± 1.45 (-2.72, 3.09)	0.90	0.032
BMI, kg/m^2^	23.32 ± 1.40	23.36 ± 1.06	-0.05 ± 0.32 (-0.69, 0.60)	0.88	0.032
% whole body fat, %	18.35 ± 4.77	18.27 ± 4.74	0.09 ± 1.16 (-1.81, 2.85)	0.66	0.017
Arm muscle mass, kg	6.06 ± 0.83	5.00 ± 0.77	1.06 ±0.21 (0.64, 1.47)	< 0.01	1.324
Leg muscle mass, kg	18.13 ± 1.80	18.12 ± 1.56	0.01 ± 0.44 (-0.86, 0.89)	0.98	0.006
SMI, kg/m^2^	8.14 ± 0.61	7.83 ± 0.56	0.32 ± 0.15 (0.02, 0.61)	< 0.05	0.529
% MMI, %	34.99 ± 2.63	33.55 ± 2.25	1.44 ± 0.66 (0.11, 2.77)	< 0.05	0.588

Notes: ^a^Values are the mean ± standard deviation or ^b^mean ± standard error. Abbreviations: % MMI = percentage of muscle mass index; % whole body fat = percentage of whole body fat; BMI = body mass index; CI = confidence interval; SMI = skeletal muscle index.

Table 4 shows the muscle strength and physical performance results for the two groups after completing the weight reduction program. Except for the handgrip strength per arm muscle mass, there were no significant differences in any of the variables between the groups. Although the arm muscle mass of the weight reduction group was significantly lower than that of the reference group, no significant difference was observed with regard to the handgrip strength between the groups. The handgrip strength per AMM in the weight reduction group was significantly higher than that in the reference group (P < 0.01). The upper extremity muscle mass decreased significantly after completion of the weight reduction program, but it did not induce an undesirable decrease in the upper extremity muscle strength. 

**Table 4:** Characteristics of the muscle strength and physical performance parameters, and the differences between the two groups after a 12-week weight reduction program.

	Reference group^a^	Weight reduction group^a^	Mean difference^b^	P	Effect size
	(n = 29)	(n = 31)	(95% CI)		
HGS, kg	44.30 ± 6.13	41.68 ± 5.56	2.62 ±1.52 (-0.43, 5.67)	0.91	0.448
HGS/Weight, kg	0.64 ± 0.08	0.61 ± 0.10	0.03 ± 0.02 (-0.01, 0.08)	0.14	0.331
HGS/AMM, kg^c^	7.35 ± 0.71	8.46 ± 1.27	-0.12 ± 0.27 (-1.66, -0.57)	< 0.01	1.079
IMT60 PTQ, Nm	175.34 ± 32.36	185.24 ± 33.27	-9.91 ± 8.56 (-27.05, 7.24)	0.25	0.302
IMT60 PTQ/weight, Nm/kg	2.54 ± 0.40	2.69 ± 0.46	-0.15 ± 0.11 (-0.38, 0.07)	0.18	0.348
IMT60 PTQ/LMM, Nm/kg	9.67 ± 1.36	10.24 ± 1.68	-0.57 ± 0.40 (-1.37, 0.23)	0.16	0.373
IKT60 PTQ, Nm	155.14 ± 28.61	160.51 ± 23.23	-5.37 ± 6.76 (-18.90, 8.17)	0.43	0.206
IKT60 PTQ/weight, Nm/kg	2.25 ± 0.38	2.33 ± 0.30	-0.08 ± 0.09 (-0.26, 1.00)	0.38	0.234
IKT60 PTQ/LMM, Nm/kg	8.56 ± 1.30	8.86 ± 1.00	-0.29 ± 0.30 (-0.89, 0.31)	0.33	0.259
IKT60 TW, J	439.03 ± 75.03	460.09 ± 65.79	-21.06 ± 18.36 (-57.82, 15.70)	0.26	0.298
IKT60 TW/weight, J/kg	6.37 ± 1.05	6.67 ± 0.84	-0.3 ± 0.25 (-0.80, 0.19)	0.23	0.316
IKT60 TW/LMM, J/kg	24.25 ± 3.42	25.41 ± 2.98	-0.15 ± 0.83 (-2.82, 0.52)	0.17	0.362
IKT60 AP, W	96.11 ± 19.69	100.63 ± 17.03	-4.52 ± 4.78 (-14.10, 5.05)	0.35	0.246
IKT60 AP/weight, W/kg	1.40 ± 0.28	1.46 ± 0.24	-0.07 ± 0.07 (-0.20, 0.07)	0.33	0.230
IKT60 AP/LMM, W/kg	5.31 ± 0.94	5.56 ± 0.80	-0.25 ± 0.23 (-0.70, 0.21)	0.28	0.286
Jump test, cm	45.04 ± 8.94	44.16 ± 6.45	0.87 ± 2.02 (-3.16, 4.91)	0.67	0.114

Notes: ^a^Values are the mean ± standard deviation or ^b^mean ± standard error; ^c^Mann-Whitney U test was employed. Abbreviations: CI = confidence interval; HGS = handgrip strength; AMM = arm muscle mass; IMT60 PTQ = isometric60 peak torque; LMM = leg muscle mass; IKT60 PTQ = isokinetic60 peak torque; IKT60 TW = isokinetic60 total work; IKT60 AP = isokinetic60 average power.

Tables 5 and 6 present the results of the one-year follow-up assessment, and the differences between the weight reduction group and the reference group. No significant differences were observed between the two groups with regard to any of the variables. 

**Table 5:** Characteristics of the subjects in the anthropometric and body composition analyses, and the differences between the two groups a year after the completion of a weight reduction program.

	Reference group^a^	Weight reduction group^a^	Mean difference^b^	P	Effect size
	(n = 29)	(n = 31)	(95% CI)		
Age, year	49.96 ± 10.45	48.71 ± 8.19	1.25 ± 3.20 (-5.22, 7.72)	0.70	0.133
Height, cm	172.23 ± 5.56	170.87 ± 6.20	1.35 ± 1.89 (-2.47, 5.18)	0.48	0.231
Weight, kg	69.21 ± 6.11	70.20 ± 5.07	-0.99 ± 1.90 (-4.82, 2.85)	0.61	0.176
BMI, kg/m^2^^c^	23.32 ± 1.40	24.02 ± 0.47	-0.7 ± 0.29 (-1.30, -0.11)	0.11	0.670
% whole body fat, %	18.35 ± 4.77	17.63 ± 3.30	0.73 ± 1.42 (-2.15, 3.60)	0.61	0.176
AMM, kg	6.06 ± 0.83	6.01 ± 0.60	0.05 ± 0.25 (-0.45, 0.55)	0.84	0.069
LMM, kg	18.13 ± 1.80	18.67 ± 1.51	-0.54 ± 0.56 (-1.67, 0.60)	0.34	0.325
SMI, kg/m^2^^c^	8.14 ± 0.61	8.45 ± 0.42	-0.3 ± 0.16 (-0.63, 0.02)	0.13	0.592
% MMI, %	34.99 ± 2.63	35.18 ± 1.85	-0.19 ± 0.79 (-1.78, 1.40)	0.81	0.

Notes: ^a^Values are the mean ± standard deviation or ^b^mean ± standard error. Abbreviations: CI = confidence interval; BMI = body mass index; % whole body fat = percentage of whole body fat; AMM = arm muscle mass; LMM = leg muscle mass; SMI = skeletal muscle index; % MMI = percentage of muscle mass Index.

**Table 6:** Characteristics of the muscle strength and physical performance parameters, and the differences between the two groups a year after the completion of a weight reduction program.

	Reference group^a^	Weight reduction group^a^	Mean difference^b^	P	Effect size
	(n = 29)	(n = 14)	(95% CI)		
HGS, kg	44.30 ± 6.13	41.25 ± 6.13	1.05 ± 2.03 (-3.06, 5.16)	0.61	0.171
HGS/Weight, kg	0.64 ± 0.08	0.62 ± 0.10	0.02 ± 0.03 (-0.04, 0.08)	0.45	0.221
HGS/AMM, kg	7.35 ± 0.71	7.23 ± 0.99	0.12 ± 0.27 (-0.42, 0.66)	0.66	0.139
IMT60 PTQ, Nm	175.34 ± 32.36	194.36 ± 33.51	-19.03 ± 10.72 (-40.69, 2.63)	0.08	0.577
IMT60 PTQ/weight, Nm/kg	2.54 ± 0.40	2.78 ± 0.52	-0.25 ± 0.14 (-0.54, 0.05)	0.10	0.517
IMT60 PTQ/LMM, Nm/kg	9.67 ± 1.36	10.49 ± 2.08	-0.82 ± 0.53 (-1.90, 0.26)	0.13	0.467
IKT60 PTQ, Nm	155.14 ± 28.61	160.73 ± 31.45	-5.59 ± 9.68 (-25.15, 13.96)	0.57	0.186
IKT60 PTQ/weight, Nm/kg	2.25 ± 0.38	2.30 ± 0.45	-0.05 ± 0.13 (-0.32, 0.22)	0.72	0.120
IKT60 PTQ/LMM, Nm/kg	8.56 ± 1.30	8.65 ± 1.72	-0.09 ± 0.47 (-1.04, 0.87)	0.86	0.059
IKT60 TW, J	439.03 ± 75.03	461.90 ± 89.67	-22.88 ± 26.25 (-75.93, 30.18)	0.39	0.277
IKT60 TW/weight, J/kg	6.37 ± 1.05	6.60 ± 1.25	-0.23 ± 0.37 (-0.97, 0.51)	0.54	0.199
IKT60 TW/LMM, J/kg	24.25 ± 3.42	24.83 ± 4.75	-0.58 ± 1.28 (-3.16, 2.00)	0.65	0.140
IKT60 AP, W	96.11 ± 19.69	102.38 ± 21.83	-6.27 ± 6.68 (-19.78, 7.23)	0.35	0.302
IKT60 AP/weight, W/kg	1.40 ± 0.28	1.46 ± 0.31	-0.07 ± 0.09 (-0.26, 0.12)	0.48	0.203
IKT60 AP/LMM, W/kg	5.31 ± 0.94	5.51 ± 1.19	-0.2 ± 0.34 (-0.88, 0.48)	0.65	0.187
Jump test, cm	45.04 ± 8.94	46.43 ± 6.35	-1.39 ± 2.68 (-6.81, 4.02)	0.61	0.179

Notes: ^a^Values are mean ± standard deviation or ^b^mean ± standard error; ^c^Mann-Whitney U test was employed. Abbreviations: CI = confidence interval; HGS = handgrip strength; AMM = arm muscle mass; IMT60 PTQ = isometric60 peak torque; LMM = leg muscle mass; IKT60 PTQ = isokinetic60 peak torque; IKT60 TW = isokinetic60 total work; IKT60 AP = isokinetic60 average power.

## DISCUSSION

This pilot study aimed to investigate whether weight reduction significantly decreases muscle mass, muscle strength, or physical performance to cause health problems, and the primary findings of this study were as follows: First, the AMM in the weight reduction group was significantly less than that in the reference group after completion of the weight reduction program. Low AMM contributed significantly to the low SMI and %MMI in the weight reduction group compared with that of the reference group. Second, there were no significant differences between the two groups with respect to the absolute or relative (strength per body weight and muscle mass) values for muscle strength and physical performance, except in the handgrip strength per AMM, which was significantly higher in the weight reduction group than in the reference group. Third, no significant differences between the groups were observed for any of the variables after a one-year weight reduction program. This result suggested that weight reduction resulting from a combination of caloric restriction and an exercise program did not induce an undesirable decline in muscle mass, muscle strength, or physical performance. These findings are inconsistent with existing reports that weight reduction can cause an undesirable decrease in muscle mass and strength, which is, thus, likely to decrease physical performance ^11^^,^ ^26^. 

It has been reported that weight reduction programs consisting of exercise do not cause a significant decrease in leg muscle volume and leg muscle strength ^27^. However, those consisting of caloric restriction are linked to significant decreases in leg muscle volume and strength ^27^. In addition, decreases in fat-free mass and the cross-sectional area of leg muscle induced by weight reduction programs consisting of caloric restriction and exercise are less than those induced by weight reduction programs consisting of caloric restriction alone ^28^. Based on the results of these previous studies, it is recognized that exercise during caloric restriction is expected to minimize decreases in muscle mass and strength. However, all of these previous studies evaluated changes in muscle mass and strength immediately after the completion of a weight reduction program. The studies did not investigate whether weight reduction leads to detrimental decreases in muscle mass, muscle strength, or physical performance that could lead to health problems. 

The results after the completion of the weight reduction program indicate that there were no significant differences between the reference and weight reduction groups with respect to total body fat percentage and LMM, whereas the AMM, SMI, and %MMI in the weight reduction group were significantly lower than those in the reference group. The low SMI and %MMI in the weight reduction group were derived from the low AMM. Low AMM after the completion of a weight reduction program might be a cause of concern. However, there was no significant difference between the reference and weight reduction groups with regard to handgrip strength, and the handgrip strength per AMM in the weight reduction group was significantly greater than that in the reference group. Absolute muscle strength is the easiest way to measure muscle strength, whereas relative muscle strength (muscle strength per body weight or muscle mass) might be more relevant for indicating functional impairments ^29^^,^ ^30^. Muscle strength per muscle mass has been widely employed to determine muscle quality ^30^^-^^32^. Thus, our results indicate that the muscle quality in the arm muscle mass improved after completion of the weight reduction program. The absolute and relative (strength per body weight and muscle mass) values of leg muscle strength in the weight reduction group were not significantly lower than those in the reference group. Therefore, it is difficult to conclude whether muscle mass, muscle strength, or physical performance decreased inappropriately. 

Previous studies have recommended resistance exercises to regain or increase lost muscle mass and strength induced by weight reduction on the basis that ground-reaction forces and related rates of loading during physical activity are increased in combination with a decline in muscle mass and strength ^8^^,^ ^9^^,^ ^13^^,^ ^14^^,^ ^26^. Resistance exercises to regain or increase muscle mass and strength might not be required, because a year after the completion of the weight reduction program in our study, muscle mass and strength returned to the preweight reduction state. In addition, Santanasto et al. ^33^ reported that compared to a weight reduction program consisting of exercise alone, one consisting of caloric restriction and exercise further improved physical function because the thigh fat area decreased 6-fold relative to the lean area after weight reduction. Beavers et al. ^34^ reported that a change in fat mass is a more significant predictor of changes in physical function than a change in lean mass is. Weight reduction decreases the physical burden on the musculoskeletal system and does not cause an inappropriate decrease in muscle mass and strength. These changes are very likely to contribute to an improvement in physical performance. 

This study has two limitations. First, the conclusion of this study was based on the results of a weight reduction program consisting of caloric restriction and exercise. It remains unclear whether a similar conclusion could be drawn after the completion of a weight reduction program based only on caloric restriction. Further research is required to confirm whether weight reduction programs with caloric restriction induce an undesirable decrease in muscle mass, muscle strength, or physical performance. Second, we could not perform the follow-up assessment in the reference group. Future research will benefit from a study protocol that includes a follow-up assessment for the reference group. 

## CONCLUSION

The quality of the upper extremity muscles in the weight reduction group was significantly better than that of the muscles in the reference group, and no significance was detected between the two groups with regard to the other variables at the completion of the weight reduction program. A year after the completion of the program, no significant differences between the groups were found in any of the variables. Therefore, the results of this pilot study suggest that weight reduction with caloric restriction and exercise does not induce a detrimental decrease in muscle mass, muscle strength, or physical performance. 
